# Editorial: Lactate metabolism and regulation of the immune response

**DOI:** 10.3389/fimmu.2022.1103379

**Published:** 2022-12-09

**Authors:** Marcela Hortová-Kohoutková, János G. Filep, Jan Frič

**Affiliations:** ^1^ International Clinical Research Center, St. Anne’s University Hospital, Brno, Czechia; ^2^ Department of Pathology and Cell Biology, Université de Montréal, Montreal, QC, Canada; ^3^ Department of Modern Immunotherapy, Institute of Hematology and Blood Transfusion, Prague, Czechia

**Keywords:** lactate, inflammation, immunometabolism, immune cell, immunoregulation

It was our pleasure to have a chance to review several fascinating articles on the topic addressing the role of lactate metabolism in the immune response. This synopsis summarizes the main perspectives described within each of the accepted articles in this collection.

In spite of the ground-braking discovery of Otto Heinrich Warburg in the 1920s, within the immune system research, the role of lactate has been overlooked for a long time. Nevertheless, with the better and detailed understanding of immunometabolism, the important regulatory role of lactate is being increasingly recognized. Lactate is currently perceived not only as an important biomarker but also as a critical energy source for mitochondrial respiration, a signaling molecule or even a precursor of other metabolic processes. Indeed, a strong emphasis has been given to dissecting the role of lactate in the tumor microenvironment and recently described the role of lactate in the regulation of long-term cellular reprogramming through a process called lactylation. The articles in this collection led further insights into these events.

Chen et al. in their article entitled “*Lactylation, a Novel Metabolic Reprogramming Code: Current Status and Prospects*”- summarizes the novel molecular mechanisms by which lactate regulates the actual and even long-term changes of cellular metabolism and energy control. The findings are interestingly discussed in the context with cancer progression and drug resistance. In particular, the article provides several important examples of how lactate fine-tunes the complexity of disease pathology by acting as a regulator of overall metabolism, immune reactions, and intercellular communication resulting in lactate-mediated reprogramming of immune cells and enhancement of cellular plasticity contributing to disease-specific immunity status.

Manoharan et al. discussed lactate as an important signaling molecule. Their review “*Lactate-Dependent Regulation of Immune Responses by Dendritic Cells and Macrophages*” addresses again the diverse roles of lactate in the control of inflammation and other regulatory responses in various tissues under homeostasis and pathological conditions, such as autoimmunity or cancer. These authors discussed immune cell subsets, predominantly macrophages and to some extent, dendritic cells as among the most intensively studied cells in terms of immunometabolism, as profound metabolic changes are crucial for the maintenance of innate immune response during infection or inflammation. While emphasizing the key aspects of lactate in the biology of macrophages and dendritic cells, the authors provide a basis for understanding lactate-controlled processes in other cell types. This article together with that of Jedlicka et al. provides deeper insights into the direct control of immune functions regulated directly by lactate.

Further connection of lactate present in the tissue or even tumor microenvironment has been presented by Jedlicka et al. in the paper entitled “*Lactate from the tumor microenvironment - A key obstacle in NK cell-based immunotherapies*”. The authors provide an overview how lactate in the tumor microenvironment would lead to polarization of phenotypes of dendritic cells and more importantly how lactate may impair the cytotoxic properties of CD8+ T cells and NK cells. This article also highlights lactate as one of the potential obstacles to the immune response. This would raise the clinically relevant question of therapeutic targeting of lactate levels as adjuvant therapy in some scenarios.

The conclusions of lactate’s importance in the immune response processes are further corroborated by the article of Jimenez-Duran et al. by showing that lactate is essential to control the complement function. An important connection between the lytic machinery of complement and the release of lactate was clearly demonstrated in the original paper of Jimenez-Duran et al. entitled “*Complement membrane attack complex is an immunometabolic regulator of NLRP3 activation and IL-18 secretion in human macrophages*”. Thus, challenging human monocyte-derived macrophages with membrane attack complex resulted in profound metabolic changes, including release of lactate into the extracellular space. While the authors describe lactate release as a by-product of other stimulation and processes, other articles of this collection clearly underscore the important role of extracellular lactate in shaping the tissue microenvironment within the inflamed site.

Changes in lactate levels have been shown to be associated with a large number of disorders. The number of publications addressing lactate in the control of the immune system function is rising every year. This Research Topic providing a collection of original and review papers clearly illustrates the vibrancy of the field and hopefully will provide some ground for further application of advancing our understanding of the important role of lactate.

## Author contributions

MH-K, JGF, JF wrote and revised the editorial. All authors contributed to the article and approved the submitted version.

